# Urinary incontinence is associated with the development of peptic ulcers in adult women: Data from the KNHANES IV

**DOI:** 10.1097/MD.0000000000008266

**Published:** 2017-10-27

**Authors:** Jin Bong Choi, Byung Il Yoon, Kyung-Do Han, Sung-Hoo Hong, U-Syn Ha

**Affiliations:** a Department of Urology, Bucheon St. Mary's Hospital, College of Medicine, The Catholic University of Korea, Bucheon; b Department of Urology, International St Mary's Hospital, The Catholic Kwandong University of Korea, Incheon; c Department of Biostatistics; d Department of Urology, Seoul St. Mary's Hospital, College of Medicine, The Catholic University of Korea, Seoul, Republic of Korea.

**Keywords:** peptic ulcer, psychological, stress, urinary incontinence

## Abstract

The aim of this study was to investigate the association between urinary incontinence (UI) and peptic ulcer (PU) and how this is related to psychological stress in Korean women by analyzing the data from the Korea National Health and Nutrition Examination Survey IV (KNHANES).

A nationally representative sample of data on 7475 Korean women ≥19 years of age from the KNHANES 2008 to 2010 was included. Physician-diagnosed UI and PU were assessed using questionnaires and surveys. Psychological stress was assessed through a questionnaire using a 4-point Likert scale. Data were analyzed using logistic regression to determine the association between UI and PU according to the level of psychological stress perception.

PU was found in 1.41% of the total population. Breaking this down by the existence of UI, PU was found in 3.5% of the population with UI, and 1.4% of the population without UI, which showed a significant difference. A statistically significant trend for increasing prevalence of UI and PU with increasing psychological stress perception levels was found among the study population. Multivariable logistic regression analyses for PU showed that UI was significantly associated with a higher probability of PU in an adjusted model, which means that members of the population with UI were more likely to have PU than those without UI. A higher level of psychological stress perception was also significantly associated with increased odds of PU in the adjusted model.

UI could potentiate the development of PU through increasing levels of psychological stress perception.

## 1 Introduction

Urinary incontinence (UI), defined as any involuntary leakage of urine, is a major health issue for women.^[1]^ The prevalence of UI in women is reported to be as high as 55%.^[2]^ This condition not only diminishes their quality of life (QoL) but also causes problems with personal hygiene and increased economic expense.^[3,4]^ The relationship between psychological stress and female voiding dysfunction including overactive bladder or urgency UI has been described in some reports. Many patients with overactive bladder or urgency UI reported psychological stress, somatic symptoms, and lower QoL.^[5–7]^

Psychosocial stress can increase the risk of stroke, heart disease, and digestive problems. Gastrointestinal functions such as peristalsis, digestion, defecation, and reflux inhibition are controlled by the complex autonomic neurohumoral system. Emotional stress may influence this system, and induce irritable bowel syndrome, gastro-duodenal ulcers, gastro-esophageal reflux disease, etc.^[8]^ Among these digestive problems, peptic ulcer (PU) is a relatively common problem, present in about 4.1% of the population, which can cause severe complications such as gastrointestinal bleeding and perforation.^[9,10]^ It therefore remains an important issue in health. Although *Helicobacter pylori* infection and nonsteroidal anti-inflammatory drugs are known to cause most cases of PU, about 20% of patients with PU have no identifiable organic etiology.^[11,12]^ An increase in ulcer disease after natural calamities such as earthquakes has been well described and documented. In these patients, psychosocial factors including emotional stress may play a role in triggering PU disease.^[13,14]^

Previous studies have focused only on the correlation between psychological factors and PU, or the association between UI and psychological stress. To our knowledge, no study investigated these 3 factors in the same sample of women. Therefore, the objectives of this study were to examine the correlation between the prevalence of UI and psychological stress, evaluate the correlation between psychological stress and the incidence of PU, and investigate the association between UI and PU in Korean women.

## 2 Materials and methods

The Korea National Health and Nutrition Examination Survey (KNHANES) is a cross-sectional survey of a nationally representative sample of the Korean population. Data used in this study were acquired from the KNHANES IV (2007–2009). It consists of health interviews, health examinations, nutrition surveys, and laboratory investigations. The study population for this analysis included adult women more than 19 years old who participated in all 3 parts (UI, PU, and psychological stress) of the survey. Each subject's identification number was anonymized for protection of the individual's privacy.

Subjects were categorized as having PU if they replied “Yes” to the question, “Do you have physician-diagnosed peptic ulcer?” Psychological stress was estimated from answers indicative of cognitive complaints obtained with a questionnaire applying a 4-point Likert scale as follows: very severe, severe, moderate, low. Subjects were classified as suffering UI if they replied “Yes” to the question, “Do you have physician-diagnosed urinary incontinence?” Patents with previous PU or UI who had been cured were not included. The definitions of lifestyle, socioeconomic, and clinical variables have been entirely described in previous studies.^[15,16]^

**Figure 1:**
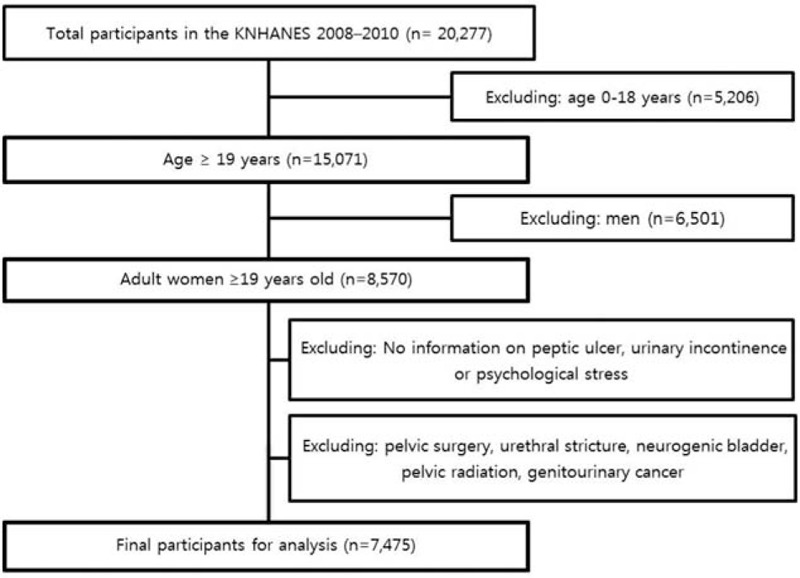
Study design and disposition of subjects.

Of the 20,277 participants who underwent the KNHANES IV, we initially selected 8570 adult women ≥19 years old. Subjects who were missing data on PU, UI, or psychological stress were excluded. In addition, subjects with a history of pelvic surgery, neurogenic bladder, urethral stricture, pelvic radiation, and genitourinary cancer were also excluded. After excluding ineligible subjects (n = 1095), the final study group included 7475 women (Fig. 1). The percentage of missing values was about 10% for the variables. Sensitivity analysis was used to handle missing data. This study was approved by the Institutional Review Board of the Catholic University of Korea (No. KC15EISI0502). Anonymized and de-identified information was used for analysis, and therefore, the need for informed consent was waived.

The statistical analysis was carried out using SAS version 9.2 (SAS Institute Inc., Cary, NC). The data in this study are expressed as the mean ± standard error (SE) or percentages (SE) for continuous or categorical factors, respectively. The prevalence of PU and UI were analyzed according to psychological stress perception. Multivariate adjusted logistic regression analysis was conducted to examine the odds ratio (OR) and 95% confidence interval (95% CI) for the association between PU and UI according to the psychological stress perception level. Calculations were made adjusting for age, body mass index (BMI), smoking, alcohol drinking, exercise, highest diploma, UI, psychological stress perception, metabolic syndrome, diabetes, hypertension, and menopause status. A *P* value < .05 was considered statistically significant.

## 3 Results

PU was found in 1.41% (106/7475) of the total population. PU was found in 3.5% of the population with UI and 1.4% of the population without UI, which showed a significant difference (*P* < .0001). Table 1 presents the general characteristics of the group according to presence of PU. Exercise, residential district, highest diploma, and household income were significantly different between the populations with and without PU. Table 2 summarizes the clinical characteristics of the group according to presence of PU. The prevalence of UI was much higher in the population with PU than those without PU. The mean age, BMI, and waist circumference (WC) were significantly higher in those with PU than in those without PU. Of those with PU, hypertension, metabolic syndrome, and menopause were significantly more frequent than in those without PU. In addition, participants with PU were likely to report more psychological stress perception in comparison with their counterparts.

**Table 1:**
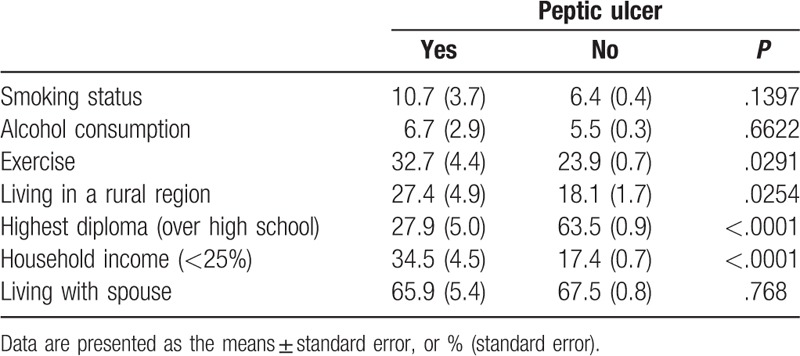
General characteristics of the subjects according to the existence of peptic ulcer.

**Table 2:**
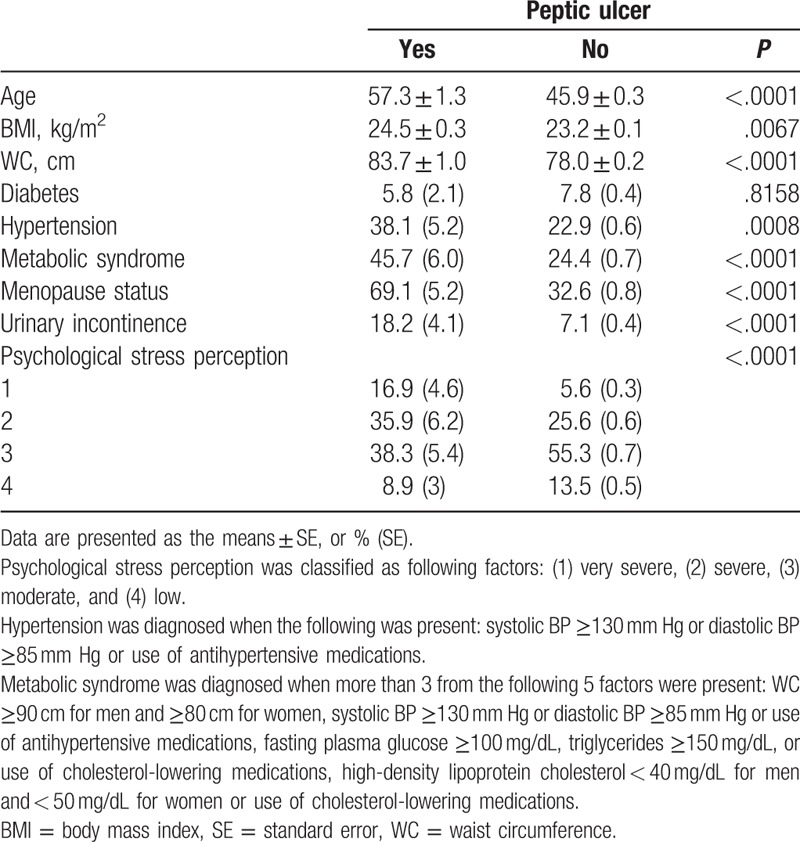
Clinical characteristics of the subjects according to the existence of peptic ulcers.

**Figure 2:**
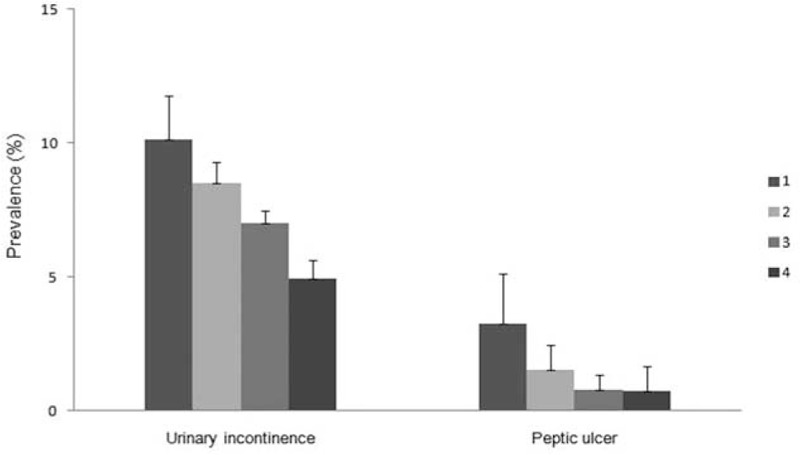
Prevalence of peptic ulcer and urinary incontinence according to the level of psychological stress perception: (1) very severe, (2) severe, (3) moderate, (4) low.

A statistically significant trend for increasing prevalence of UI (*P* = .0022) and PU (*P* = .0002) with psychological stress perception was found among the study population (Fig. 2). The prevalence of UI was lowest (4.9%) in those with level 4 psychological stress perception and highest (10.1%) in those with level 1 psychological stress perception. Similar trends could be identified in the prevalence of PU, which was lowest (0.7%) in those with a psychological stress perception of 4 and highest (3.3%) in those with a psychological stress perception of 1.

Multivariable logistic regression analyses for PU (Table 3) show that UI was significantly associated with a higher probability of PU in an adjusted model, which means that those with UI were more likely to have PU than those without UI. Higher psychological stress perception was also significantly associated with increased odds of PU in the adjusted model.

**Table 3:**
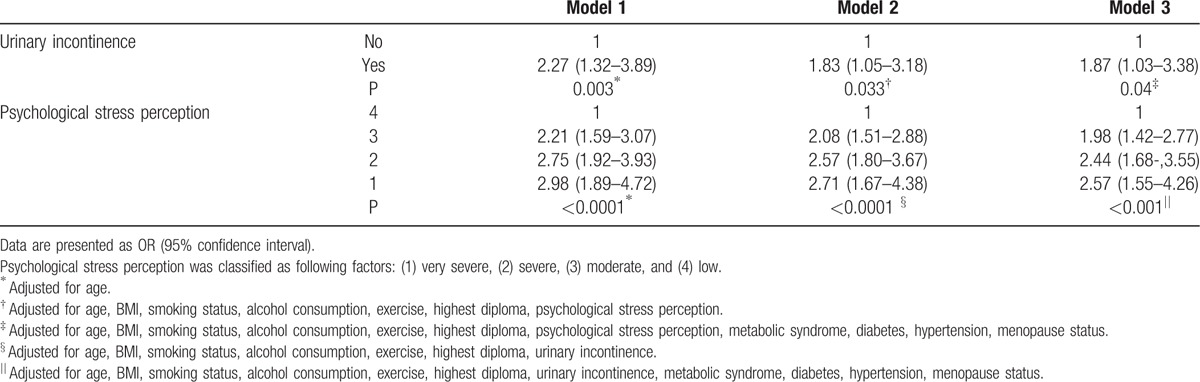
Adjusted ORs of peptic ulcer according to urinary incontinence and psychological stress perception.

## 4 Discussion

The main findings of this study are as follows: PU was found more frequently in women with UI than in women without UI; Individuals who display higher psychological stress perception levels that are indicative of elevated psychological stress are more likely to have UI and PU; and UI and psychological stress perception were significantly associated with a higher probability of PU in an adjusted model. On the basis of these finding, we can speculate that UI could potentiate the development of PU in accompaniment with increasing psychological stress perception.

The underlying pathophysiology for these outcomes can be explained by the association between psychological distress and UI. Several reports suggest psychological distress secondary to UI.^[17–20]^ De Vries et al^[19]^ suggest that women with UI were more likely to undergo new psychological stress than women without UI (relative odds, 2.18). Henry et al^[6]^ also reported that there was a positive correlation between perceived stress level and UI. Recently, a large population-based study reported that UI was associated with an increased risk for onset of depression and work disability.^[20]^ Several reports have provided evidence that UI and psychological health have common biochemical or neurological pathways, such as serotonergic functioning,^[21–23]^ and further neurochemical abnormalities underlying both incontinence and psychological morbidity may imply a common genetic defect.^[21]^

Although the association between psychological stress and PU is controversial, recent reports^[14,24]^ lend weight to the role of psychological stress in developing PU. In a recent prospective study, psychological stress increased the risk for PU, in part by influencing health risk behaviors.^[14]^ Stress had similar effects on ulcers associated with *H. pylori* infection and those unrelated to either *H. pylori* or the use of nonsteroidal anti-inflammatory drugs.^[14]^ These clinical results are supported by biological studies showing that stress may promote PU through increased acid load, the effects of hypothalamic-pituitary-adrenal axis activation on healing, altered blood flow, or cytokine-mediated impairment of mucosal defenses.^[25–27]^ These reports linking UI with psychological distress and subsequent development of PU could partially account for the underlying pathophysiology of our results.

The noticeable point of this study is that it is the first nationally representative population-based study to suggest that UI can be a potential risk factor for PU among Korean adult women who participated in KNHANES 2007 to 2009. UI not only causes urinary tract symptoms but also other morbidity including psychological disease. Even though a few studies on the relationship of UI to mental health and QoL have been conducted,^[28]^ there is little study of the impact of psychological distress secondary to UI on organic disease. These findings provide new information and motivation for further research about the association between UI-related psychological distress and other organic diseases. However, we should always consider the relative prevalence rate difference between urinary symptoms and other organic diseases.

There are some limitations on this study. First is the definition of UI, from self-reported questionnaires, which was defined as a yes answer to the question “Do you have physician-diagnosed urinary incontinence?” To overcome limitations caused by the quality of the responses, which may be impaired due to memory recall and individual reasons of the participants, we enrolled only participants with physician-diagnosed UI. UI is divided into 3 major subtypes by the International Continence Society: urgency incontinence, stress incontinence, and mixed incontinence. Psychological stress secondary to UI can differ depending on the subtype of UI. We could not supply a subanalysis according to these 3 subtypes, because this information is not recorded in KNHANES. However, considering that several studies that investigated the impact of UI on mental health found no significant differences among the subtypes of UI, although less than 30 patients were targeted,^[29,30]^ we carefully hypothesized that psychological stress is correlated to urine leakage symptoms rather than to the subtype of UI. This limitation therefore does not seem to affect our results.

The other limitation is that we cannot fully explain the underlying pathophysiology of PU and UI. As mentioned above, these results could be partly explained based on previous reports, but there is currently not enough evidence to explain these results clearly. The relationship between PU and psychological stress is not yet clear, despite recent evidence lending weight to the role of psychological stress. Psychological stress may trigger or potentiate PU. Likewise, PU may give rise to psychological stress. There also might be an unknown common pathophysiology related to both PU and UI. Regardless, the result that PU was more frequently found in women with UI is worthy of notice. Paying attention to the impact of UI-related psychological distress can help us to better understand what is needed to improve management of UI.

Lastly, the KNHANES IV did not include detailed information about *H. pylori* infection and nonsteroidal anti-inflammatory drug use. Thus, we could not evaluate the impact of those factors and future high-quality studies are needed.

## 5 Conclusion

Our study reported for the first time that PU was more frequently found in women with UI than in those without UI. UI and an increased perception of psychological stress were significantly associated with a higher probability of PU. On the basis of these findings, we can speculate that UI could potentiate the development of PU through increasing psychological stress perception. These findings show that physicians who care for patients with UI also need to manage the psychological distress seen with this condition.
